# Practical guide to structuring the Methods and Results sections in clinical and epidemiological studies

**DOI:** 10.1186/s12998-026-00644-0

**Published:** 2026-09-01

**Authors:** Claire Gudex, Charlotte Leboeuf-Yde

**Affiliations:** 1 Research Unit OPEN, Department of Clinical Research, University of Southern Denmark, Odense, Denmark; 2 Department of Regional Health Research, University of Southern Denmark, Odense, Denmark

**Keywords:** Writing process, Structure of a scientific article, Methods section, Results section

## Abstract

This is the second of three articles that provide step-by-step guides on how to structure the content of a scientific article to ensure a coherent and relevant text. This article deals with the Methods and Results sections. We start by explaining the purpose and content of these sections and then provide a practical guide for planning the structure of each section as preparation for writing the full text later. Thus, we describe ten steps for structuring the Methods section and six steps for structuring the Results section. We illustrate our explanations with text examples selected from this journal to show how authors have approached various aspects of these two sections. After some tips for constructing informative tables and figures, we discuss the use of artificial intelligence when preparing the Methods and Results sections. The Methods section is fundamental to your reader understanding your study and trusting the validity of your findings, while the Results section provides answers to your stated research objectives or questions. Transparency and completeness are essential in both sections to assist your reader and reflect your academic integrity.

## Introduction

This is the second of three articles on how to structure a scientific article to produce informative and easily read reports of clinical and epidemiological studies. The current topic is the Methods and Results sections. The first article deals with the Background section [1], and the third with the Discussion section [2].

Here, we first discuss the purposes of the Methods and Results sections and then present an approach for determining the structure and content of each section. Finally, we discuss the possible uses (or not) of artificial intelligence (AI) in preparing the Methods and Results sections. We illustrate many of the points using examples taken from articles published in this journal.

## Why do we have Methods and Results sections?

The Methods section consists of a full description of everything that was done to obtain the study results. There are four main purposes behind this:

- To enable your study to be reproduced by others (a basic principle of research is that any study should give the same or similar results if repeated in exactly the same way).
- To show that you have conducted the study in an ethical way, i.e. that you have taken the necessary precautions to safeguard your study participants and the confidentiality of their data; that you are transparent and honest about your data collection and analysis.
- To show the validity of your methods, i.e. that your chosen study design fits with your research objectives and that your methodological approach covers the research objectives and is suitable for that type of study design.
- To indicate how you have maximized the quality of the data collected and have minimized the risk of bias in your findings.

In other words, the Methods section is where your readers can understand how you have carried out your study and decide whether they should trust your study findings.

The Results section builds on the Methods section by reporting the findings of your study. It describes the characteristics of the study sample and provides answers to each of your research objectives in turn. This description of your results must be both transparent and complete.

*Transparency* means that the results for all research objectives can be easily identified, the data variables are clearly labelled and defined, and the number of observations (also for subgroup analyses) is easily seen for each analysis. Notably, no results must be left out because they were unexpected or provided ‘negative’ findings. If one of the research objectives could not be investigated, the most transparent approach would be to keep it in the list of research objectives and explain why it could not be dealt with.

*Completeness* means providing all the necessary details to describe the study sample, being clear about how each analysis was done, presenting the findings for all the research objectives, and presenting results from any additional activities used to validate or test data.

## Before planning your Methods and Results sections

You do not need to have collected and analysed your data before structuring your Methods and Results sections. However, you do need to have determined your research objectives (and overall aim, if relevant) as far as possible so that you have an idea about how you will go about your study. You will also need to have a good idea of several other aspects of your study, such as the frequency of data collection and your variables of interest—see Text box 1 for more details.

Text box 1. Information needed before planning the content of your Methods and Results sections

- Your research objectives
- The study design you will be using
- Any relevant ethical considerations
- The source for your study sample
- Recruitment of your study sample, whether done by you or previously by others
- Whether an intervention is included or you will be dividing your study sample into comparative groups for another reason
- The frequency of data collection on or from your study sample
- The techniques, tools, equipment, etc. you will use to collect or measure the data on each variable
- Your variables of interest (i.e. the dependent/outcome variable and any independent (or other) variables for each research objective) and how you will use these variables (e.g., reporting how common something is, how something develops over time, how one variable affects others, or differences between estimates from different interventions)
- Whether you need to define any conditions or terms, describe classifications, etc.
- Relevant aspects of data quality and/or bias

## What information to include – and what not to include

The text of the Methods and Results sections must be fully informative. For the *Methods section*, this means a detailed description of all the methodological choices and actions related to your research objectives. The Methods section is often quite long and a bit boring! To avoid long and technical descriptions of tests or procedures, you can move details of these into an additional file or perhaps just provide a reference for the reader to obtain more detailed information. Very specific information can also be referred to as “available from the authors on request”.

The content of the Methods section will differ according to the study design that has been chosen. Different study designs have different requirements in terms of e.g., the study population, data collection and analysis, and ethical considerations. This makes it difficult to describe how to write a typical Methods section. Nevertheless, the Methods sections of clinical research projects will include aspects of the following items:

(i) Description of the study design and ethical considerations.
(ii) Intentions regarding the study sample (e.g., source, recruitment strategy, selection criteria).
(iii) Possibly a description of intervention(s) (e.g., treatment and control activity, allocation procedures).
(iv) Description of what data were collected, when and how.
(v) Maximization of data quality.
(vi) Description of how the data were analysed.

A fully informative *Results section* means that it builds on the Methods section by reporting the findings for all your research objectives. The Results section should not include information about how the study was undertaken (that text belongs in the Methods section), nor any judgment or interpretation of the results, e.g., “to our surprise, we found…” (that text belongs in the Discussion section). The Results section should also not include any information that is not covered by the research objectives! If you have ‘extra’ findings, you probably need a further research objective.

A typical Results section consists of three to five items, depending on the study design:

(i) The size of the final study sample relative to the invited/expected study sample (e.g., sample size, response rate, adherence to protocols, compliance with data collection).
(ii) A description of the final study sample (characteristics, representativeness).
(iii) Any preliminary information (e.g., results of a pilot or feasibility study, a reliability test, or the success of blinding in an RCT).
(iv) Answers for each research objective.
(v) Any additional findings such as a post-hoc analysis (e.g., adding an extra analysis on existing data to better understand a result).

## Order of information in Methods and Results sections

For the Methods section, it is usually easiest for the reader if you describe your actions in chronological order (i.e. the order you did them in real life) and do not jump back and forth. For example, you need to explain who your study population was before you can describe what data you collected from them or what tests you did on them. If it better suits your research objectives, however, you can choose a different order, e.g. by variable (the most important first) or in the order of the research objectives (if these differ greatly from each other). The use of headings will help the reader understand your logical approach to this.

## Some tips for ordering the information in the Methods section:

- Imagine your colleague wants to repeat your study, and you have to explain what to do step by step—and by telephone!
- Write down these brief instructions in the order that would make most sense for your colleague.

## Some tips for ordering the information in the Results section:

- After describing your final study sample (and perhaps adding some information to show whether the data can be trusted), just follow the same order as your research objectives!
- Your readers do not want to have to search through your text for information – make it easy for them by presenting the results in the expected order and using informative headings.

## A practical guide to structuring your Methods section

The following steps guide you through the process of structuring your Methods section, advising you to use short notes to indicate what needs to be written in more fully later. At the end, you will have produced a list of headings with notes underneath that provides the outline for the whole section and describes your methods logically. You and your co-authors can then use this outline to check that you have included each relevant step and that the order of information makes sense and has the necessary links to the research objectives. When you are confident that your study methods will not change, you can start to fill out the notes with full sentences.

### Check your research objectives

First, look carefully at your research objectives and make sure that they clearly explain all your research activities and that they all fit under the same study design. The name of the research design may be included as an introduction to the research objectives (which are usually located at the end of the Background section or at the very beginning of the Methods section).

### Make a list of headings

List your six or seven headings, as relevant for your study: (i) *Description of the study design;* (ii) *Ethical considerations*, (iii) *Information about obtaining the study sample*, (iv) *Any interventions or control groups*, v) *Description of what data were collected*,* when and how*, vi) *Data quality*,* and* vii) *Description of how the data were analysed.* Thereafter, add brief notes of information under each of these headings. Table 1 provides examples of headings in the Methods section of three published articles with different study designs—a descriptive cross-sectional study, a prospective observational study, and a randomized controlled trial. Table 2 provides suggestions for the notes under each heading in the cross-sectional study.

**Table 1:** Examples of headings that can be used to structure the Methods section

Descriptive cross-sectional study [3]	Prospective observational study [4]	Randomized sham-controlled trial [5]
Study design and setting	Study design and setting	Trial design
Recruitment and participants	Participants - Eligibility criteria - Procedures	Participants
Data collection	Treatment	Interventions - Multimodal rehabilitation protocol - Trigger point diagnosis - Experimental group - Control group
Cox^®^ Flexion Distraction procedure	Variables - Clinical outcome measures - Improvement criteria - Adverse events - Candidate prognostic baseline factors o Individual’s factors o Clinical factors o Therapist-related factors	Outcomes Primary outcome measure - Resting pain Secondary outcome measures - Passive and active range of motion - Shoulder pain and disability index, Persian version - Strength in shoulder movements
Ultrasound measurements	Statistical methods	Sample size - Sequence generation
Statistics		Blinding and concealment
		Statistical methods

**Table 2:** Suggestions for notes that could indicate the content under each heading in a Methods section

Headings in the Methods section of Kruse et al. [3]	Our suggestions for the notes under each heading
Study design and setting	Study design Location of study
Recruitment and participants	Convenience sample Type of population Method of recruitment Time of recruitment Inclusion/exclusion criteria Ethical considerations
Data collection	Positioning during measurements Timing of measurements Reference to origin of protocol Positioning of ultrasound probe Determination of degree of flexion
CFD procedure	Performed by two licensed clinicians Description of procedure Reference to usefulness of treatment protocols Degrees of flexion varied between individuals
Ultrasound measurements	Ultrasound equipment identified Description of procedure Reference to reliability of method
Statistics	How demographic data were reported Normality assessment Determination of significance Use of t-test Use of linear regression Software used

### Description of the study design

Under this heading, you will have to think about how much information you need to provide. This is going to be the reader’s introduction to the whole Methods section. If the study is fairly simple or straightforward and the study design is clear from the research objectives, you may just need to provide a few words to indicate the study design and population (e.g., a randomised single-blind clinical trial, a descriptive study using the (NAME) register, a qualitative study using focus groups among patients with…), and how many times data were collected. If your project is a complicated one, however, it may be a good idea to start the Methods section with a ‘mini-introduction’, i.e. a few sentences that indicate the overall design of the study, e.g., the timing of data collections (e.g., baseline and follow-ups), the comparisons made (e.g., before-and-after analysis on the same study participants, use of intervention and control groups), or how the different parts of the study are linked. It may even be helpful to provide a figure or table showing, for example, the timing of different data collections, how the variables of interest relate to each other, the links between the different steps in the study. Table 3 provides examples of ‘mini-introductions’ in the Methods section of three published articles with different study designs—a descriptive cross-sectional study, a prospective observational study, and a qualitative study.

**Table 3:** Examples of ‘mini-introductions’ found in the Methods section of three articles

Study design	Text describing aspects such as study design, reporting guidelines, and summary description of the study
Cross-sectional study	“This study employed a descriptive cross-sectional study design, which aimed to explore publicly available information regarding LBP on the websites of South African chiropractors as well as its alignment with evidence-based practices. This study was conducted and reported in accordance with the STROBE guidelines for observational studies [22.23].” [6]
Prospective observational study	“In this prospective observational exploratory study, we gathered candidate prognostic baseline factors (i.e. factors collected before and during the first visit) from patients presenting a new episode of NSLBP and attending either one of two chiropractic institution outpatient clinics (CC) or one of the nine affiliated chiropractic offices (CO) in France between March 1, 2022, and February 28, 2023. …This article follows the recommendations of the REMARK checklist [14].” [4]
Qualitative study	“This study adhered to the Standards for Reporting Qualitative Research (SRQR) guidelines [27] and employed interpretive description [28–30] as its qualitative research approach, with a constructivist lens. This approach facilitates the exploration of chiropractors’ experiences in the delivery of rehabilitation to equity-deserving groups in Canada, yielding insights for addressing health disparities.” [7]

### Ethical considerations

Write notes for the various consents and approvals you need to mention (including if you did not need ethical permission according to national laws), and anything else about data confidentiality or storage. Note the use of any register data and refer to previous publications if you are conducting secondary analyses—and document the permission to use these data that have been collected by others, if relevant. Journals usually require additional ethical aspects under supplementary information for the article, such as trial registrations, author contributions, funding, and conflicts of interest. Table 4 provides examples of different approaches to reporting ethical considerations due to specific study circumstances.

**Table 4:** Examples of different approaches to reporting ethical considerations due to specific study circumstances

Study circumstances	Examples
Clearance from ethics committee when ethical permission was not needed	Both examples are from Supplementary Information (under ‘Declarations’): “This study did not report on or involve the use of any animal or human data or tissue and received ethical clearance from the University of Johannesburg Research Ethics Committee (REC-2577-2024).” [6] “The Regional Committees on Health Research Ethics for Southern Denmark determined (reference number S-20140050) that, according to Danish regulations, the Danish Chiropractic Low Back Pain Cohort did not require ethical approval. All participants gave written informed consent to participate.” [8]
Secondary analysis of previously published study with reference to previous report	Within text: “Our study is a secondary analysis of data from the Back Complaints in Elders – Chiropractic (BACE-C) study, a 12-month observational, longitudinal cohort study of older adults (> 55 yrs) with LBP presenting for chiropractic primary care [23] in the Netherlands, Sweden and Australia.” [9]
Multicentre study needing permissions in several countries	In Supplementary Information (under ‘Declarations’): “All research groups in the BACE-C international consortium have obtained ethics approval for their national study, separately. The Netherlands study received ethical approval from the Medial Ethics Committee of the Vrije University Medical Center (approval number 207–618). The Swedish study received ethical approval from the Karolinska Institute (approval number 2018/474 − 31/2).The Australian study received ethical approval from the Macquarie University Science & Engineering Subcommittee. Human Research Ethics Committee (project ID 5460) and from the CQUniversity Human Research Ethics Committee (approval number 023408).” [9]
Clinical trial	Within text: “The UMN’s Institutional Review Board approved the study (STUDY00013265) and all participants provided electronic consent for screening and study enrollment. An independent monitoring committee reviewed and monitored the study.” [10] In Supplementary Information (under ‘Declarations’): “The research reported in this report has been performed in accordance with the Declaration of Helsinki. Ethical approval for the study was provided by the Institutional Review Board at the University of Minnesota (STUDY00013265). All participants provided consent to participate.” [10]

### Information about obtaining the study sample

Add notes here about what you need to explain. It may be relevant to first present the source of the study sample (e.g., persons from the general population fitting certain criteria, all patients with a special condition attending a hospital department, whether you collected your data yourself or got them from a database, register, or earlier project), and you may place your definition of the disease/condition here. Then, it would probably be appropriate to explain how you obtained your study sample through the recruitment or identification process. Items you may need to explain are random (or other approach) selection, the inclusion/exclusion criteria, sample size requirements, and the dates for recruitment or inclusion in the study. When structuring your manuscript, most of these pieces of information can be made into subheadings with short notes underneath to remind you what you will write out in full later. Table 5 provides examples of how different articles described the study population and how the study sample was selected.

**Table 5:** Examples of how articles described (a) study population and (b) selection of study sample

Description of study population	Description of how the study sample was selected
[Patients attending] “Shafa Yahyaian Hospital’s physiotherapy clinic, Tehran, Iran.” [5]	“…patients with a history of rotator cuff tendon tear that had undergone open RCR surgery in Shafa Yahyaian Hospital…The inclusion and exclusion criteria are shown in Table 2.” [5]
“…patients…attending either one of two chiropractic institution outpatient clinics (CC) or one of the nine affiliated chiropractic offices (CO) in France between March 1, 2022, and February 28, 2023.” [4]	“Consecutive patients with a new episode of NSLBP aged 18 years or older were enrolled. A new episode of NSLBP was defined as having pain or an increase of pain present for more than 24 h and preceded by a month without pain or stable pain [15]. The exclusion criteria were the following: ….” [4]
“…generally healthy volunteers of all sexes and ethnicities, aged 21 years or older, who denied having LBP…” [3]	“The participants were recruited through announcements and flyers that were posted on the Keiser university campus from May 2022 to June 2022. Exclusion criteria included ….” [3]

### Any interventions or control groups

You may need many separate notes here to remind you of what to write later as you will have to describe all the procedures undertaken, e.g., group allocation procedures (random or otherwise, concealed or not), and a description of the intervention/control activities including a presentation of the persons who performed the various interventions (e.g., number, qualifications, prior training) and instructions/calibration of activities. The control group(s) may receive another type of treatment, a sham/placebo treatment, or no treatment at all. Groups may be parallel or consecutive, or the control may be historical. Any blinding must be described (for the personnel at randomization, treatment, assessment and/or analysis; and for the study subjects).

### Description of *what* data were collected, *when* and *how*

Your notes here need to reflect all the steps taken in the data collection process, and these steps might differ according to your variables of interest. At this stage, you don’t need to worry about the ordering of the information but just describe each data collection or each variable in turn. You will likely need to first explain techniques or equipment used to measure or collect the data on each variable, and then to explain which variables you extracted (e.g., from a questionnaire or a set of clinical tests), and how the data for each variable were interpreted or coded (e.g., cut-off points for levels of severity, histological grading, coding of questionnaire responses, measurement units). Remember to note whether the data have been collected or analysed for a single timepoint (cross-sectional) or across several timepoints (e.g., time series or longitudinal study) and the dates of data collection. If these data were collected by somebody else (e.g., register study, secondary analysis of previous study), you must still explain their origin and list the variables you retained for your present report. Table 6 provides examples from Methods sections showing how authors separated the description of data collection from the description of variables used in the analysis.

**Table 6:** Examples from Methods showing how description of data collection is reported separate from description of analytical variables

Description of data collection	Description of variables used in the analyses
Hansen et al. [8] Questionnaires at baseline on iPad in clinic. Follow-up questionnaires sent by e-mail after 2,13, and 52 weeks.	All variables of interest were listed in text under separate subheadings, describing the instruments used (with references) or the questions/calculations used. Variables: age and sex, disability, pain, psychological factors, social factors, body mass index, comorbidities, analgesics.
Hadizadeh et al. [11] Primary outcome variables: Goniometer Ultrasound Secondary outcome variables: VAS Digital algometer Neck disability index Ultrasound Color Doppler ultrasonography	Cervical range of motion (ROM) Circumference of trigger points Pain intensity Pain pressure threshold Neck disability Longitudinal and transverse diameters, and stiffness of trigger points Blood circulation
Galaasen Bakken et al. [12] Numeric rating scale and a short-form McGill questionnaire Neck disability index EQ-5D-3L Clinicians used their journal notes	Pain intensity, quality, and effective components of pain Neck disability Health-related quality of life Number and dates of treatments, and the treatment modalities used in each case.

### How data quality was maximized

Now consider (i) the quality of your data (necessary to obtain correct information) and (ii) the potential for bias (systematic error that might under- or overestimate your findings). In most Methods sections, quality is ensured by using e.g., interview guides, calibrated equipment, trained staff, double data entry, transparent data analysis, and processes for dealing with missing data. Bias is avoided by, e.g., using an appropriate selection process, complete databases or registers, dealing with missing data, a proper random selection or allocation process, and blinding of examiners, study subjects, and statisticians. Go back over the list of headings and notes that you have made in the above steps. For each note in turn, consider if there could be a danger of poor data quality and/or a risk of bias. Add any extra notes under the relevant item about what this danger or risk was and what you did to (try to) prevent it.

### How the data were analysed

There are five points here:

(i) Note preliminary statistical information such as data storage, data cleaning, how to deal with missing data, description of variables in the analyses (including transformation of variables), power calculation if not earlier in the Methods section.
(ii) Write down the type of analysis that each research objective leads up to, e.g., descriptive as percentages; prevalence/incidence estimates; between-group differences as odds or risk ratios; comparisons of treatment outcomes; between-group differences in change over time as in RCTs.
(iii) Add the names of statistical tests, e.g., t-test, linear regression.
(iv) Indicate the level of statistical significance.
(v) Name the software used.

Table 7 shows different approaches for reporting data management and statistical analyses in the Methods section. For a more detailed guide on how to describe the statistics for different types of analyses, see Lang and Altman [13].

**Table 7:** Examples of different approaches for reporting aspects of data management and statistical analysis

Aspects covered	Examples
Pre-hoc sample size calculation	“We assumed an anticipated overall outcomes proportion around 50% based on previous studies in older adults with back pain [28,29], we expected an acceptable C-statistic of at least 0.7 [30], we set the standard error (SE) for C-statistic, calibration slope and ‘calibration-in-the-large’ less than 0.025, 0.15 and 0.15, respectively [27], and applied the R package, sampsizeval [27]. This resulted in an estimated sample size of at least 428 participants.” [9]
An example of data management	“Data was primarily collected using electronic data capture through REDCap, a secure web application for building and managing online surveys and databases [71]” [10]
Rare reporting of unbiased statistical analysis	“The statistical analysis was performed using STATA Statistics version 13.0 (which was performed by somebody who was not involved in the study process)” [11]
Two examples of the analysis of descriptive data	“Baseline characteristics of participants were summarized using means and standard deviations (SD) for continuous variables and frequencies with percentages for categorical variables.” [4] “Descriptive data for the whole population and for each age group (< 40, 40–59, and ≥ 60) were presented as proportions for categorical variables and as means and confidence intervals, or median and interquartile interval (IQI) for continuous variables depending on the distribution of data.” [8]
Checking the normality of data	“Data normality was checked for all continuous variables using the Shapiro-Wilk test, histogram, and skewness and kurtosis.” [5]
Two examples of how to perform simple comparisons The second example continues with an explanation of a more complex analysis between variables	“We used independent t-test for continuous variables with a normal distribution and Mann-Whitney U test for not normally distributed data, and chi-square test for categorical variables to compare the demographic variables and pain characteristics among the different SBT risk subgroups.” [9] “To determine significance, statistical paired t-tests were used to compare the spinous process distances before the manipulation loading and during application of the Cox technique. An independent t-test was conducted to identify any statistically significant differences between male and female participants. Additionally, linear regression analyses were carried out to examine the relationships between body mass index (BMI) and the change in separation distance, as well as between age and the change in separation distance.” [3]
Descriptions of univariate and multivariate regression, specifying the purpose of the analyses and the variables included	“Univariate linear regression analyses were performed to examine the relationship between disability scores (dependent variable) at 2,13 and 52 weeks follow-up and age group (independent variable). Afterwards a multivariate linear regression model with all potential predictive variables were [sic] performed at each of the follow-up times to examine the relationship between disability and each of the potential predictive variables, adjusted for age group.” [8]
Specifying the purpose of AN(C)OVA and the types of variables included	“The analysis of variance and covariance (ANOVA/ANCOVA) was used to determine between group differences of all continuous data while the pre-intervention values of outcomes were included as a covariate and groups as a factor (one factor, one covariate; primary analysis)” [5]
A brief report of the analysis of qualitative data with references to justify the approach	“A rapid deductive, directed content analysis was conducted for qualitative data from open ended survey questions and the post-study focus groups interview with providers. Rapid approaches can balance rigor with efficiency, yielding timely and meaningful evaluation of stakeholder perspectives [74,75]” [10]
Reporting level of statistical significance	“*P*-values smaller than 0.05 were considered statistically significant" [12]
Two detailed examples of dealing with statistical and clinical significance	“The level of significance was set at 0.05. In addition, the point estimates of effects were presented as MD with 95% confidence interval (CI) and standardized mean differences (SMD) with 95% CI, analyzed using Cohen’s d.” (an interpretation of Cohen’s d effect size followed this text) [5] “The point estimates of effects were reported as MD with a 95% confidence interval (CI), standardized mean difference (SMD) with 95% CI analyzed using Glass’s Delta. The statistical significance level was determined as *p* < 0.05.” (an interpretation of Glass’s Delta effect size followed this text) [11]
Dealing with missing data	“We used multiple imputation with five imputed datasets and 10 iterations [31] to handle the missing values for predictors and outcomes. Complete case analyses were performed as a sensitivity analysis.” [9]

### Review the structure of your Methods section

Now you can review the order of your headings and their notes. Imagine you are a reader who is seeing these study methods for the first time. Do you think the order of information makes sense, or should some information be moved—perhaps to a different heading or even to a heading of its own? Note that the information on ethics usually comes either at the start or the end of the Methods section. In this way, the ethical considerations cover the whole of your methodological approach.

Most journals require authors to use a relevant reporting guideline and to complete a checklist of content. These reporting guidelines are useful for identifying items that you may have omitted in your own list (see 'Reporting Guidelines' at the end of this article). Finally, check the content of your Methods section using the tips in Text box 2.

Text box 2. Tips for checking the content once you have structured your Methods section

- Look at each of your research objectives in turn:
  - For the variables of interest, have you described where you obtained the data? How the data were collected, and how they were interpreted or coded? Measurement unit? Information on data quality and risk of bias?
  - For the specified purpose of each research objective, have you explained how the variables were quantified or compared and how they are presented (frequencies, means, etc.), and is each statistical test named?
- Are any of your research objectives not covered in the Methods section? If so, you need to add this information to your Methods section.
- Are there any statistical tests mentioned in the Methods section that are NOT represented by a research objective? If so, you probably need to add a further research objective.
- If your Methods section would be scrutinized in a systematic review, have you sufficiently covered quality and potential risk of bias issues for your study to be considered methodologically sound? (You can check this by applying an appropriate Risk of Bias checklist).

## A practical guide to structuring your Results section

The steps below guide you through the process of structuring your Results section.

### Make a list of the relevant subheadings

Select the subheadings you think you might need from this list:

- Sample size/response rate.
- Description of study sample.
- Any additional information (e.g., pilot/feasibility study, reliability test, success of blinding).
- Results relating to your research objectives.
- Any post-hoc analyses.

### Sample size/response rate

Collect the relevant numbers and percentages for sample size or response rate and write these in. You must always report the number (proportion or response rate) of people who were invited to join the study, the number who entered the study, and the number of participants at each follow-up point. Make sure that you use the correct (initial) numerator at the subsequent follow-ups if there are several follow-ups, not just the previous one! If you have intervention and control groups, provide both numbers and percentages in each group and at each timepoint. If any of this information is complicated, show the participants on a flowchart—this is required for randomized controlled trials (CONSORT flowchart). If the response rate is unknown, you should state this, e.g., if you recruited patients from a hospital clinic but did not know the total number of patients attending the clinic or the number who declined participation. Table 8 provides examples of how sample size and response rate were reported in the text of the Results section.

**Table 8:** Examples of how sample size and response rate were reported in the Results section

Information reported in text of Results section	Examples	Comments
Only reported numbers (sample size) and proportion of study participants (response rate)	“We included data from 1803 participants across eight randomized trials. Complete clinical outcome and cost data was available for 1488 (83%) of participants.” [14]	Cost-effectiveness study. No flowchart needed.
Reported numbers and proportion of participants at follow-ups, summarized from a flowchart	“Among the 241 participants included, 34 (14%) did not respond to any follow-up questionnaires. A total of 194 participants (80%) completed the 7-day follow-up questionnaire, and 166 (69%) completed the 4-week follow-up questionnaire.” [4]	
Reported numbers of potential participants and final sample	“A total of 2,848 participants were available at baseline. After excluding 71 due to missing RMDQ scores, the final sample consisted of 2,777 participants, of whom 1,066 were categorized in the young group (< 40 years), 1,270 in the middle-aged group (40–59 years), and 441 in the older group (> 60 years).” [8]	The response rate was not reported, but it can be calculated from the sample sizes provided.
Response rate and sample size with supporting CONSORT flowchart	“44 participants were recruited. Of these, 14 were excluded, including 10 because they met the exclusion criteria and 4 because of refusal to participate. Therefore, 30 patients were included; 15 were assigned to the IMES group and 15 to the DN group. No participants dropped out during the study (Fig. 6).” [11]	It would be possible to remove some of this information from the text as it is easily found in the flowchart.
Only reported the number of participants who were interviewed in the current qualitative study BUT the number of potential participants was explained in the Methods section	Results: “Fourteen participants were recruited and participated” Methods: “A convenience sample… was recruited as part of a cross-sectional survey… (n = 3143, 41% response rate). Those who completed the study were asked… if they were interested in participating in a qualitative interview (n = 282)… a random sample of 155 individuals was contacted, and 14 participants were ultimately included in the study.” [7]	The number of potential participants and response rates could alternatively have been reported in the Results section.

If you are uncertain whether the sample size text belongs to the Methods or Results section, consider that:

- The Methods section states the intended sample size and sample characteristics and describes how you intended to achieve them (e.g., recruitment approach, power calculation, inclusion and exclusion criteria).
- The Results section reports any information you obtained after the start of data collection, i.e. the size and characteristics of the final study sample achieved. Therefore, the sample size and response rates should be placed in the Results section.

Occasionally, however, the final sample size is known when planning the study. This happens in some qualitative interview studies or when the current study uses data collected previously. In that case, this information can be provided in the Methods section—as long as any further case selection is described in the Methods and then presented in the Results section. For example, in the Methods section: “The data for the current study came from a database of 130 patients with diabetes (reference), from whom we selected adult patients with type 2 diabetes”. And in the Results section: “Our selection process resulted in a study sample of 65 adults with type 2 diabetes”.

### Description of the study sample

List all the variables you will use to describe the study sample. Your reader needs to know the main characteristics of your sample. This is partly to allow comparison with their own patient groups or the samples in previously published articles, but also to understand who the participants are. The findings of most studies will be influenced by sample characteristics such as age, sex, educational level, geographic location, severity of disease, so this information is important to present.

If you have only two or three sample characteristics to report, you can just provide these data in the text. However, studies usually have descriptive or baseline information about clinical status and medical history as well as other biological or physiological variables. These are usually best reported together in a table (typically 'Table 1') to provide an easy overview of the data and are often presented for various subgroups as well as for the total study sample. The 2017 article by Pickering [15] provides a helpful guide for presenting descriptive statistics in the text and tables.

### Results relating to your research objectives

Copy your research objectives in here, for your own use, to ensure that you will present the results of all the relevant analyses in this same order, one by one. Consider keeping relevant text from the research objectives as subheadings and reporting the results for each of them clearly separated from each other. This will ensure that you do not forget to provide any results that should be reported and prevents you from reporting on analyses that you had not planned on doing! It also makes it easier for the reader to find the results of the separate objectives.

For each of your research objectives in turn, determine the main results that you will present and write down relevant notes or key words. The results can be reported either in text only or in tables/figures with accompanying text that simply summarizes the main findings of the table or figure. Use ‘text only’ if the results are simple to report and easily comprehensible. If the results are a bit more complicated, use a table if the exact estimates are important, and a figure if you want to illustrate differences over time or between groups. Most journals do not allow you to present the same data as both a table and a figure, so you need to choose one of these for each result.

For non-significant findings, you don’t need to report the exact estimate in the text – just that the result was not significant. Numerical details such as confidence intervals, correlation coefficients, model fit statistics, and p-values are typically best shown in a table. When reporting percentages in the text, consider whether the decimal points make sense (“24% of participants preferred….” is probably more memorable than “23.78% of participants preferred….”).

Note that the text should not repeat the information in the tables but instead summarize and guide the reader to the main points. The reader can then look at the table or figure and gather more detailed data if desired. Table 9 provides examples of how baseline information and results from analyses shown in tables or figures can be summarized in the Results text. The article by Lang and Altman [13] provides a useful checklist for reporting the results of different types of analyses.

**Table 9:** Examples of how information shown in tables or figures were summarized in the Results text

Text summaries of information in tables or figures	Examples
Baseline information reported in general way with only few numbers. Most of the findings in the table are not repeated in the text.	“Participants were nearly equally distributed across the years of study and sex. The median age of the 19 participants was 24, with little variability (Table 1).” [16]
Two examples of how a lot of baseline information can be summarized simply and by referring to the tables. The second example shows how the reader can be referred to the table without a summary in the text.	“The participants (*n* = 241) were, on average, 44.2 (+/- 15.7) years old, mostly men (*n* = 156, 65%). Details of the baseline characteristics of the included sample are shown in Tables 2 and 3.*”* [4] “The baseline characteristics for the original intervention and control groups, as well as the comparative groups, can be found in Table 1.” [12]
Results of different analyses from a large table summarized/interpreted in few words.	“The results of within-group analysis revealed that both the experimental and control groups improved in pain, as measured by NPRS (*p* < 0001; Table 5).” “Accordingly, differences in the baseline measurements, age, and gender couldn’t affect the findings of the mentioned outcomes…(Table 5).” [5]
Results from a simple figure interpreted in one sentence	“Fig. 4 explored the correlation between separation distance and age, and revealed an R^2^ value of 0.58, indicating a similar level of fair to moderate association.” [3]
Results interpreted from a complex figure	“Participants generally had high confidence independent of whether or not they were able to maintain their preload and perform the lower peak thrust forces (200 N and 400 N). Notably, confidence levels became lower and more variable as the target peak forces increased to 800 N (Fig. 2).” [16]

### Additional analyses

Any minor, post-hoc analysis that does not require a special research objective is usually reported here, together with an explanation of why it was necessary.

Epidemiological studies and longitudinal clinical studies often have an analysis on the generalizability of the final study sample. This could be a comparison between the target sample and the final sample (e.g., age, sex, severity of illness), or a comparison between those who participated in the study at baseline and those who later dropped out. These findings are often reported just after the reporting of sample size or sample characteristics.

In clinical studies, a pilot or feasibility study is sometimes conducted before the main study to investigate whether the recruitment processes or assessment procedures are suitable. A reliability test may have been conducted to test the usefulness of the outcome variables.

These findings are often reported before the main results (of the research objectives) together with a brief description of the methods used. Alternatively, these methods and results can both be placed in the Methods section under a suitable heading or, if the text is extensive, in an additional file.

Note that a study that failed to reach the desired sample size should not be ‘sneaked’ into the literature as a ‘pilot study’ and that, typically, pilot studies should not be used to report results and do hypothesis testing. They have the specific purpose of testing whether the upcoming ‘main’ study can be carried out as planned or whether some aspects of it need adjusting.

In studies where participants are blinded to the intervention, the success of this blinding can be reported and may be placed before the main results.

Review the structure of your Results section Finally, check the content of your Results section using the tips in Text box 3.

Text box 3. Tips for checking the Results section

- Do the findings in the Results section follow the same order as in your research objectives?
- For each of your research objectives in turn:
  - Does your Results section provide a direct answer to the research objective?
  - Does the text in the Results section use the same wording for the variable(s) and associated concepts as in the research objective?
- Are there any research objectives whose findings are not reported in the Results section? If so, you need to add this information or remove the research objective(s). Obviously, you will also report negative findings.
- Are there any findings in the Results section that are NOT represented by a research objective? If so, you probably need to add a further research objective or expand on an existing one. If the result is irrelevant to the aims of the study, however, you must remove it.

## Constructing tables and figures

The construction of clear and informative tables and figures is a complicated topic, for which reason we provide only some general advice. For a detailed description of how to handle tables and figures, we recommend that you refer to the excellent article by Vickers et al. [17].

Most journal instructions to authors require that each table and figure can be understood on its own (without the full text of the article). This means that the table should be ‘self-sufficient’ based on the title (for tables) and legend (for figures), the information within the table, and the explanation of abbreviations under the table/figure. The test is whether someone unfamiliar with your study could look at one of your tables or figures and understand its main message without reading the surrounding text. Each table or figure should indicate the main participant characteristics (patients, disease), perhaps where they were recruited from, the number of participants/observations, and explain the variables presented and any abbreviations. The title or legend must, therefore, be more descriptive than you probably think is necessary. Titles such as “Descriptive table” or ”Baseline data for both groups” are unhelpful.

The number of participants in the study and in each analysis must also be transparent. You can sometimes use the title/legend for this (e.g., “The baseline characteristics of 383 women presenting with…in a study of…” or “The baseline characteristics of women presenting with… in a study of….(N = 383)”). However, if the number of observations differs between variables, it is better to place this information within the table for each variable. If the number of missing data for each variable is included within the table, ensure that the responses add up to 100%.

Be particularly careful about indicating sample size or number of observations for each of your figures. This information is often omitted and can make a figure ineffectual. In figures, the best place to indicate number of observations is in the legend.

If the journal instructions allow, you can add some of this additional information after the main title/legend (e.g. type of data presented, explanations of columns), otherwise as notes below the table or figure. Some figures include a text on interpretation of findings underneath, but this is not common when reporting clinical and epidemiological studies.

### Numbering your tables and figures

For your Methods section, review the information you wrote down when making your outline and decide whether you will use any tables or figures for any of this information. Then number them all (Table 1, Figure 1, etc.) and insert their names as memory guides in your plan. Note that you can use the same table at several points in your report. Thus, the same flowchart can be used to provide information on the research procedure in the Methods section and can then be referred to again in the Results section to show the number of participants.

Then do the same for your Results section. Review the information you wrote down when making your outline and decide whether you will use any tables or figures for any of this information. Then number them all (Table 2; Figure 2, etc.) and insert their names as memory guides in your plan.

### Sketch your tables and figures

In tables, start by determining the variables to be listed in the rows of the first column. Then think about how many columns you need (depending on which subgroups you want to present data for) and give each column an informative heading. Try to keep the tables and figures simple, but remember to indicate sample size, measurement units, types of data (means, medians, etc.) and to explain all abbreviations under the table or figure.

## Using AI for the Methods and Results sections

AI can be useful when writing the Methods and Results sections but remember the importance of adhering to ethical guidelines and being accountable for the work presented in your manuscript.

As with the Background section, you can use AI in the Methods and Results sections to improve your grammar and writing style. In the Methods section, AI might also help you to refine search strings, coding methods, or algorithms or to reword someone else’s description of a method or approach—you still need to be careful, however, as the AI might have reused text from other sources, leading to inadvertent plagiarism [18].

In the Results section, AI can help you to organize complicated data into categories or place them clearly into tables. You could also use AI to interpret your analyses, but here we would advise care. Why avoid the challenge and satisfaction of using your own intellect to consider and solve a problem after all your preceding efforts to design and conduct your project? If you spend time considering what your results mean and how they should be presented, you will get a better feel for your data and for issues you will need to discuss in your Discussion section.

Currently, we consider the dangers of using AI in your Methods and Results sections to be greater than the advantages. Watch out for these pitfalls:

- AI can make factual errors, e.g., it might change your variables’ names or make incorrect assumptions—be especially careful with abbreviations and acronyms.
- AI can create fake data or invent references that do not exist [18]—you must always verify a reference by finding the original publication.
- AI can generate nonsensical scientific diagrams and illustrations—one example led to the scientific article being retracted [19].
- As an author of a scientific article, you are required by the Vancouver authorship recommendations [20] to be accountable for all aspects of your manuscript; consider whether you can do this if you have used AI-generated data or descriptions without appropriate sources.
- Writing up your research yourself gives you extra insights into your methods and your results that may be missed if, for example, you ask AI to generate content using keywords.
- Text that you copy into an AI program may be shared with others, raising issues of confidentiality and data security.

In sum, AI can be a help but should be used under your insightful guidance and scrutiny. As noted earlier in this paper, transparency is essential in the Methods section in particular, but also in the Results section. Transparency and completeness, i.e. academic integrity, are more important here than perfect grammar or elegant text!

If you do use AI during your project or when preparing your manuscript, you should acknowledge this and explain its use. If relevant, you could provide your prompts or questions in a supplementary file [18]. The International Association of Science, Technical & Medical Publishers (STM) classification framework can help you to decide how to report your use of generative AI [21].

## Reporting guidelines

For the content and structure of the manuscript, the best writing aid is a relevant reporting guideline, and this is especially useful for the Methods section. The EQUATOR Network website (https://www.equator-network.org/) lists reporting guidelines for most study designs, e.g., observational studies, randomized trials, systematic reviews, diagnostic accuracy studies, economic studies, animal studies, and many more. In addition, the EQUATOR Network provides guidance on how to report in a range of medical specialties, including topics such as acupuncture, allergy, genetics, and nuclear medicine.

As individual studies differ, you will probably not need all the items listed in a reporting guideline, so you should use it as a guide rather than a starting point. Thus, we recommend that you first work on the content of your Methods section and then use a reporting guideline to check for any relevant items that you have omitted, and possibly to help decide the order of information. This will help ensure good alignment between your research objectives and your Methods section.

## Summary

The Methods section is fundamental to your reader understanding your study and trusting the validity of your findings, while the Results section provides answers to your stated research objectives or questions.

Underlying both the Methods section and the Results section are the research objectives. It is these that determine the study design and setting, the approach to the selection of study participants, data collection, and the choice of data analysis—and thus the type and breadth of the methods used and the findings to be reported.

The Methods and Results sections of a scientific manuscript are necessarily information-rich and are difficult to handle if you start by writing the text in full sentences from beginning to end. The structuring approach described here aims to help you get an overview of the structure and to ensure appropriate content before you start the actual writing process.

## Data Availability

No datasets were generated or analysed during the current study.

## Declarations

### Use of artificial intelligence tools

AI (Word Editor) was used to suggest spelling and grammar improvements in the text.
